# MAL protein suppresses the metastasis and invasion of GC cells by interfering with the phosphorylation of STAT3

**DOI:** 10.1186/s12967-022-03254-5

**Published:** 2022-01-29

**Authors:** Zhijun Geng, Jing Li, Sandang Li, Yueyue Wang, Lele Zhang, Qiuzi Hu, Xinru Wang, Lugen Zuo, Xue Song, Xiaofeng Zhang, Sitang Ge, Jianguo Hu

**Affiliations:** 1 Department of Central Laboratory, First Affiliated Hospital of Bengbu Medical College, 287 Chang Huai Road, Bengbu, 233001 Anhui People’s Republic of China; 2 Department of Clinical Laboratory, First Affiliated Hospital of Bengbu Medical College, Bengbu, 233001 Anhui China; 3 Department of Emergency Surgery, First Affiliated Hospital of Bengbu Medical College, Bengbu, 233001 Anhui China; 4 Bengbu Medical College, Bengbu, 233001 Anhui China; 5 Department of Gastrointestinal Surgery, First Affiliated Hospital of Bengbu Medical College, Bengbu, 233001 Anhui China

**Keywords:** GC, Invasion, Metastasis, MAL

## Abstract

Gastric cancer (GC) is the fifth leading cause of cancer-related death worldwide and is accompanied by low diagnosis and survival rates. The molecular mechanism of GC must be elucidated to improve treatment strategies. Recent research has shown that the expression of myelin and lymphocyte (MAL) protein is reduced in a variety of adenocarcinomas and has the function of suppressing tumor growth. However, the mechanism by which MAL regulates the epithelial-mesenchymal transition (EMT) in GC remains unclear. Here, we showed that MAL expression was downregulated in specimens from patients with GC and was negatively correlated with the clinical stage. Gain- and loss-of function assays showed that interference with MAL significantly increased tumor cell proliferation, metastasis, invasion and the EMT. Overexpression of MAL significantly inhibited the malignant behavior of GC cells. Moreover, MAL suppressed the malignant behavior of GC cells by inhibiting STAT3 phosphorylation in vitro and in vivo. Our data indicate that MAL suppresses the malignant behavior of GC cells via the STAT3/EMT axis. This study also provides insights into the pathophysiological process of GC and a reference for diagnosis and treatment.

## Background

Gastric cancer (GC) is the fifth most common malignant tumor worldwide, and no less than 1 million patients have been newly diagnosed with GC each year since 2018 [1, 2]. The prognosis of patients with early GC is good, but the diagnosis rate is low [3]. In clinical practice, most GC cases are diagnosed in the middle and late stages, which have a poor prognosis and a high fatality rate [4]. The onset of GC is the result of a series of changes in gene expression [5]. Therefore, exploring changes in the expression of related genes during the occurrence of GC will not only help to further clarify the pathogenesis of GC but will also provide experimental evidence for exploring potential targets of gene therapy.

Myelin and lymphocyte (MAL) protein is an indispensable membrane protein localized in lipid rafts [6]. It participates in the apical transport of proteins in polarized epithelial cells [7]. The role of its abnormal expression in tumorigenesis and development has attracted increasing attention [8]. MAL is specifically expressed in different tissues and cells, and its expression level differs in different cells of normal gastric tissue [9]. Interestingly, MAL is mainly expressed in epithelial cells of the gastric mucosa [9]. In addition, the interaction between MAL and glycosphingolipids is very important for the formation of microdomains in gastric epithelial cells. These studies reveal a key role for MAL in maintaining the normal function of the stomach.

GC is a gastrointestinal tumor derived from the mucosal epithelium that is mainly manifested by continuous pathological changes in the gastric mucosa [10]. In the present study, MAL expression in tissues from patients with GC was low; more importantly, its expression was negatively correlated with the tumor stage of the patient. We speculated that abnormal MAL expression may be an important cause of GC; MAL overexpression inhibited the invasion and migration of gastric tumor cells, and this process is regulated by the suppression of STAT3 phosphorylation.

## Materials and methods

### Materials

Patient stomach tissues were collected at the First Affiliated Hospital of Bengbu Medical College after obtaining informed consent and institutional approval. The anti-MAL antibody was purchased from Santa Cruz Biotechnology (Dallas, TX, USA). The rabbit anti-CD44 monoclonal antibody, anti-STAT3 (phospho-Y705) antibody, anti-snail + slug antibody, anti-vimentin antibody, anti-E-cadherin antibody, anti-STAT3 antibody, anti-GAPDH antibody, anti-alpha SMA antibody, horseradish peroxidase (HRP)-conjugated goat anti-mouse IgG (H + L) antibody, HRP-conjugated goat anti-rabbit IgG (H + L) antibody, Alexa Fluor^®^ 594-conjugated goat anti-mouse IgG (H + L) and fluorescein isothiocyanate (FITC)-conjugated goat anti-rabbit IgG (H + L) antibody were purchased from Abcam (Cambridge, MA, United States).

### Cell culture and treatment

The MGC-803 and HGC-27 cell lines were obtained from the China Infrastructure of Cell Line Resources. MGC-803 and HGC-27 cells were incubated in RPMI‐1640 medium containing 10% FBS, transfected with a MAL overexpression lentivirus or MAL-silencing siRNA and incubated at 37 °C in a humid chamber with 5% CO_2_.

### Immunohistochemistry

The GC tissues were fixed with 4% formaldehyde for 48 h, dehydrated, cleared, dipped and embedded in paraffin. The tissues were cut into 4 µm-thick sections and deparaffinized with xylene and a gradient of ethanol solutions. For immunohistochemical staining, the sections were incubated with 3% H_2_O_2_ for 0.5 h and blocked. Mouse anti-MAL (1:200) was added to the sections and incubated overnight at 4 °C, followed by an incubation with an HRP-conjugated secondary antibody (1:500) for 1 h at room temperature. Then, sections were developed by adding 3,3ʹ-diaminobenzidine (DAB). Finally, the nuclei were stained with hematoxylin.

### Immunofluorescence staining

For immunofluorescence staining, 0.2% Triton-100 was incubated with the sections for 15 min, and the sections were blocked. Rabbit anti-CD44 (1:400) and mouse anti-MAL (1:100) antibodies were incubated with the sections overnight at 4 °C, followed by an incubation with FITC-conjugated and Alexa Fluor^®^ 594-conjugated secondary antibodies for 2 h at room temperature. Subsequently, the nuclei were stained with 4ʹ,6-diamidino-2-phenylindole (DAPI) (Southern Biotech, Birmingham, USA), and images were captured with a confocal microscope (LSM 800, Zeiss).

### Western blotting

MGC-803 and HGC-27 cells (5 × 10^5^ cells/mL) were trypsinized and then plated in a 6-well plate. After full expansion, the cells were cultured with new basal medium and starved for 24 h. The medium was replaced with serum-free medium containing 10 μm Stattic, and the cells were incubated at 37 °C for 48 h. Total protein was extracted from the cells in each group with RIPA buffer (Thermo Scientific, Rockford, USA) supplemented with a mixture of protease and phosphatase inhibitors (Roche, Switzerland, Basel). Total protein (40 μg) was separated on SDS–PAGE gels and transferred to PVDF membranes (Millipore, Massachusetts, USA). The membrane was incubated with anti-MAL (1:1000), anti-p-STAT3 (1:1000), anti-α-SMA (1:1000), anti-vimentin (1:1000), anti-E-cadherin (1:1000), anti-snail + slug (1:1000), anti-CD44 (1:1000) and anti-GAPDH (1:1000) antibodies at 4 °C overnight. After washing, the membrane was incubated with an HRP-conjugated secondary antibody for 1 h. Finally, an imager (GE Healthcare) was used to expose the membrane and detect the bands.

### RT-qPCR

After MGC-803 and HGC-27 cells were incubated with Stattic, total RNA was extracted from the sample with the TRIzol Isolation System. Two micrograms of RNA were synthesized from each sample using the Oligo dT Primer and Random Primer First-Strand Synthesis System Kit (TaKaRa). RT-PCR was performed with the TB Green^®^ Premix Ex Taq™ II detection system (TaKaRa) according to the manufacturer’s recommendations. The comparative threshold cycle method was used to analyze the results, with GAPDH serving as an internal reference [11]. The primers used in this study are listed in Table 1.

**Table 1:** Primer sequence

Gene	Sense	Anti-sense
α-SMA	AAAAGACAGCTACGTGGGTG	AAAAGACAGCTACGTGGGTGA
VIM	GACCTGCTCAATGTTAAGATGGC	CAGAGGGAGTGAATCCAGATTAGTT
Snail	TCGGAAGCCTAACTACAGCGA	AGATGAGCATTGGCAGCGAG
Slug	CGAACTGGACACACATACAGTG	CTGAGGATCTCTGGTTGTGGT
CDH1	GATAGAGAACGCATTGCCACATA	ACCTTCCATGACAGACCCCTTAA
CDH2	ACACCATGGACAAGTTTTGGT	TGCTGTAGCGACCATTTTTCTC
CD44	ACACCATGGACAAGTTTTGGTG	TGCTGTAGCGACCATTTTTCTC
STAT3	ACCAGCAGTATAGCCGCTTC	GCCACAATCCGGGCAATCT
GAPDH	TGAAGGTCGGAGTCAACGGAT	CTGGAAGATGGTGATGGGATT

### Transwell assay

MGC-803 and HGC-27 cells (4 × 10^5^ cells/mL) were resuspended in basal medium, and 200 μL of cells were seeded into a Transwell chamber (8-μm pore size, Corning) to quantify cell migration. Then, 1 mL of complete medium was added to the lower chamber, and the cells were cultured for 48 h. Cells in the Transwell chamber were fixed with fixative for 0.5 h and stained with crystal violet dye for 0.5 h. In addition, the cells that did not pass through the polycarbonate membrane were removed, 5 fields of view were randomly selected, and the number of cells was counted.

One hundred microliters of Matrigel were evenly spread on the Transwell chamber, and the rest of the steps were performed as in the cell migration experiment above to quantify cell invasion.

### Cell proliferation assay

Cell proliferation was detected by plating 1 × 10^4^ cells/mL (100 μL/well) in a 96-well plate and cultured them in an incubator at 37 °C for 12 h. Then, 10 μL of Cell Counting Kit-8 (CCK-8) solution were added to each well and incubated at 37 °C for 3 h; the optical density (OD) value was detected with a microplate reader at 450 nm.

### Xenografts in nude mice

MGC-803 cells in which MAL was overexpressed or silenced were trypsinized and resuspended to a density of 1 × 10^7^ cells/mL in fresh medium. Then, each nude mouse was inoculated with 100 µL of the suspension in the back. After 15 days of normal rearing, the tumor masses were removed from the nude mice, measured and pathologically analyzed.

### Statistical analysis

All experiments were repeated at least three times. Data are presented as the means ± SD. One-way analysis of variance (ANOVA) and Student’s t test were used for statistical analyses. P < 0.05 indicated a statistically significant difference.

## Results

### MAL is expressed at low levels in primary tumors but is expressed at high levels in normal gastric tissues 

**Fig. 1:**
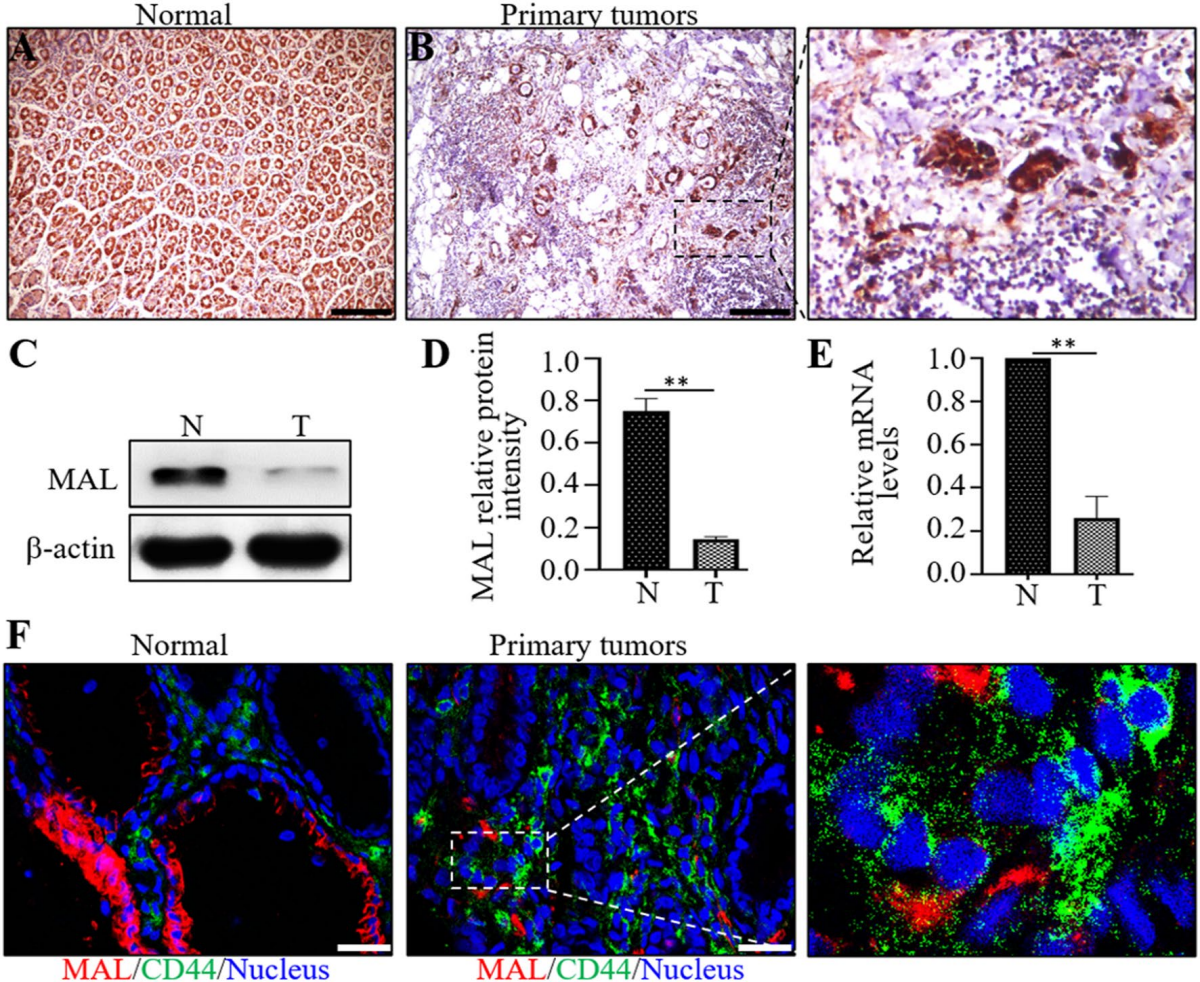
MAL is expressed at high levels in normal gastric glands but expressed at low levels in primary tumors and metastases. The expression of MAL in pericancerous (**A**) and primary GC samples (**B**) was detected using immunohistochemical staining. The expression of the MAL protein in pericancerous and primary GC samples was detected using Western blot analysis (**C**), and the statistical quantification in the two different types of GC samples is shown (**D**). The MAL mRNA expression level in the two different types of GC tissue samples was analyzed using RT-qPCR; pericancerous samples were used as the control (**E**). Confocal microscopy images show the double labeling of MAL and CD44 in pericancerous (**F**) and primary tumors (**F**). The experiments were performed at least 3 times independently, and one representative result is shown. The results are presented as the means ± SD. *P < 0.05 and **P < 0.01. A–B Scale bars = 100 μm; F scale bar = 25 μm

MAL is an indispensable membrane protein component of lipid rafts that participates in the apical transport of proteins in polarized epithelial cells [12, 13]. Here, MAL expression patterns were detected using immunohistochemistry and molecular biology analyses in pericancerous and cancerous tissue. In pericancerous tissue, high expression of the MAL protein was detected in glandular cells of the stomach (Fig. 1A). However, the expression of MAL in primary tumor tissues was substantially downregulated (Fig. 1B). We also confirmed these results by performing Western blot and RT–qPCR analyses (Fig. 1C–E).

CD44 is a cancer stem cell marker that is expressed at high levels during tumorigenesis and in epithelial cells; it is associated with a poor clinical prognosis and cancer metastasis [14]. In this study, we performed CD44 and MAL costaining in the collected GC samples to identify the level of the MAL protein in cancer cells expressing stemness markers as a method to assess the relationship between MAL protein levels and cancer stemness. In pericancerous samples, CD44 was not expressed in epithelial cells, and MAL was expressed at high levels in glands of the gastric mucosa (Fig. 1F). However, CD44 was upregulated and MAL was significantly downregulated in tumor tissues (Fig. 1F).

### MAL expression is downregulated in advanced stages of GC

**Fig. 2:**
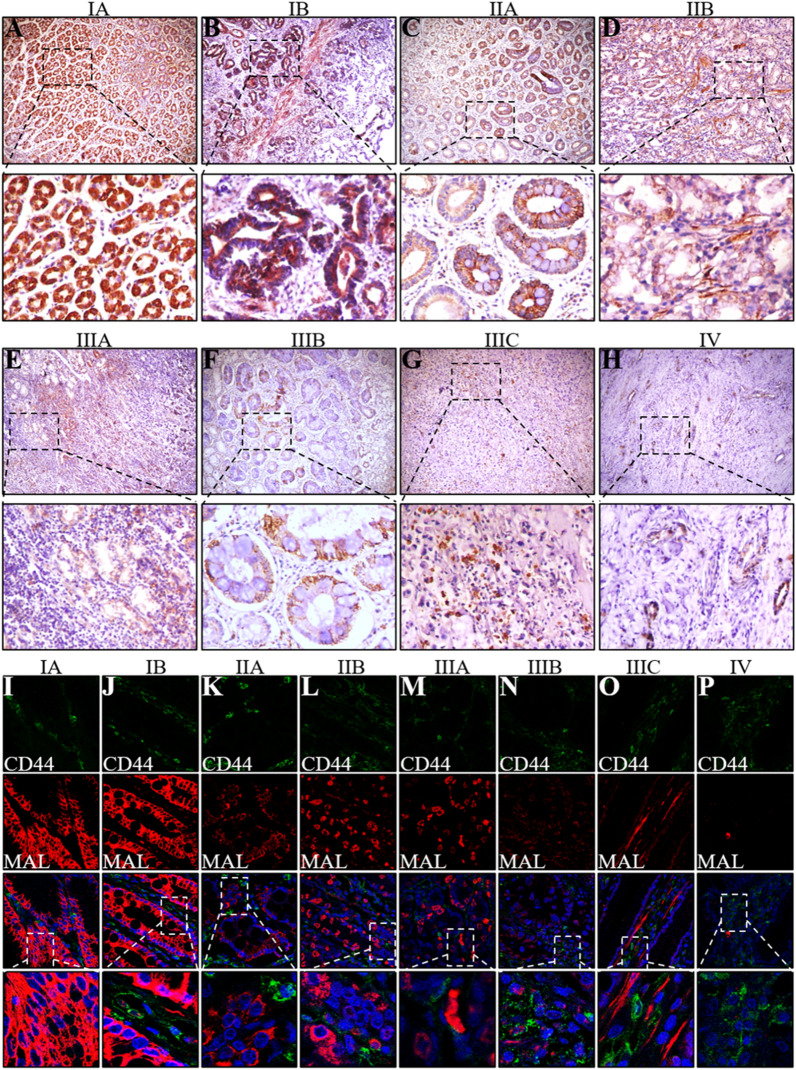
The histopathological analysis showed that MAL is expressed in primary GC tissues of different clinical stages. The expression of the MAL protein in primary GC tumors of clinical stage IA (**A**), IB (**B**), IIA (**C**), IIB (**D**), IIIA (**E**), IIIB (**F**), IIIC (**G**), and IV (**H**) was detected using immunohistochemistry. Confocal microscopy images show the double labeling of CD44 and MAL in primary GC samples of clinical stages IA (**I**), IB (**J**), IIA (**K**), IIB (**L**), IIIA (**M**), IIIB (**N**), IIIC (**O**), and IV (**P**) detected using immunohistochemistry. The experiments were performed at least 3 times independently, and one representative result is shown. A–H, Scale bars = 100 μm; I–P, scale bars = 25 μm

The interaction between MAL and glycosphingolipids is essential for the formation of microdomains in gastric epithelial cells. In addition, MAL is a tumor suppressor gene, and its hypermethylation is related to a higher survival rate of patients with GC. In the present study, we performed immunohistochemical staining of GC tissues of different pathological stages and observed high MAL expression in early GC, similar to the results obtained for normal epithelial cells, but MAL was expressed at extremely low levels in advanced GC (Fig. 2A–H). In addition, we performed CD44 and MAL costaining of these samples and found similar expression patterns for MAL; CD44, a tumor stem marker, was expressed at low levels in early GC and at high levels in advanced GC (Fig. 2I–P).

### MAL inhibits the proliferation, metastasis and invasion of GC cells

**Fig. 3:**
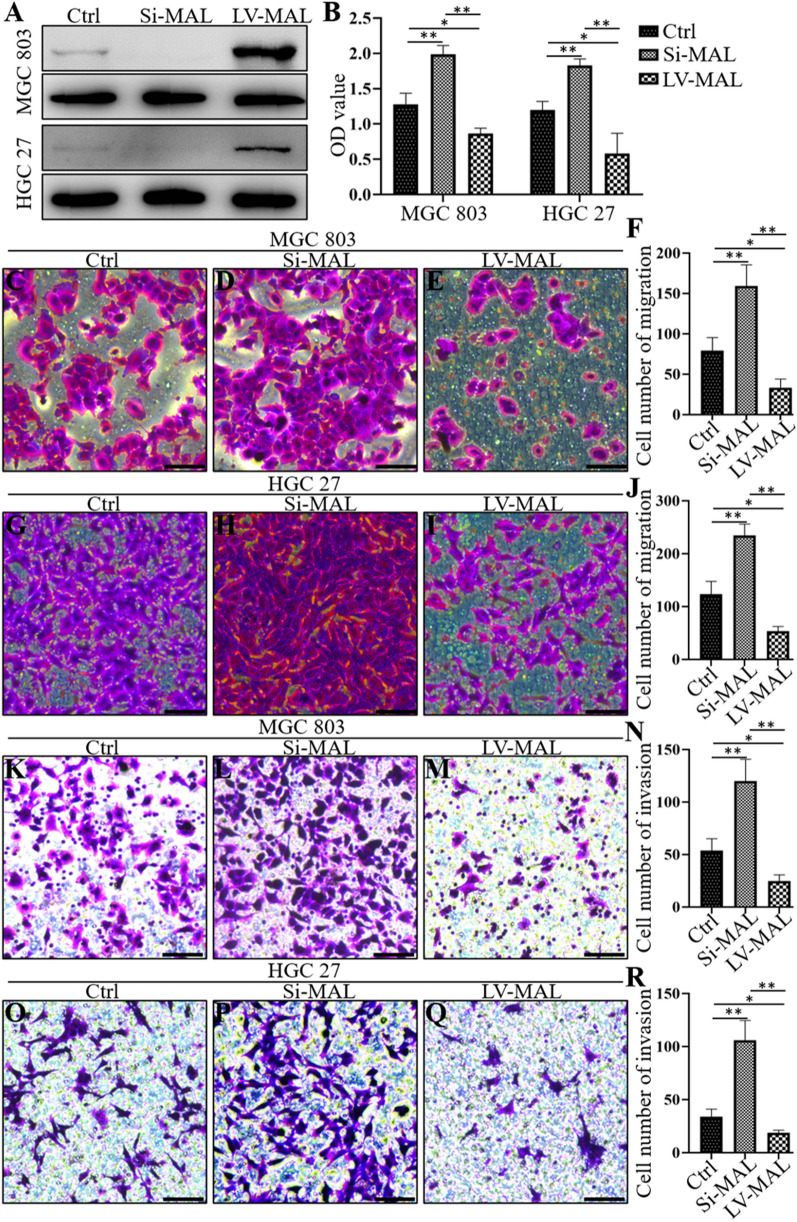
The MAL protein interferes with the proliferation, metastasis and invasion of GC cells. Western blotting confirmed that transfection of MGC-803 and HGC-27 cells with the siRNA significantly downregulated the expression of the MAL protein, while transfection with the MAL expression plasmid upregulated MAL expression (**A**). The CCK-8 results showed that MAL knockdown increased the proliferation of MGC-803 and HGC-27 cells, while MAL overexpression inhibited the proliferation of MGC-803 and HGC-27 cells (**B**). MAL silencing increased the metastasis of MGC-803 cells, and MAL overexpression reduced the metastasis of MGC-803 cells (**C**–**F**). MAL knockdown increased the metastasis of HGC-27 cells, and MAL overexpression inhibited the metastasis of HGC-27 cells (**G**–**J**). MAL silencing increased the invasion of MGC-803 cells, and MAL overexpression inhibited the invasion of MGC-803 cells (**K**–**N**). MAL knockdown enhanced the metastasis of HGC-27 cells, and MAL overexpression inhibited the metastasis of HGC-27 cells (**O**–**R**). The experiments were performed at least 3 times independently, and one representative result is shown. The results are presented as the means ± SD. *P < 0.05 and **P < 0.01. C–E, G–I, K–M and O–Q, Scale bars = 100 μm

We silenced and overexpressed the MAL gene in GC cells and verified the results by performing Western blotting to confirm the role of MAL in the occurrence and development of GC (Fig. 3A). The CCK-8 assay showed that MAL knockdown significantly increased the proliferation of MGC-803 and HGC-27 cells (Fig. 3B), while MAL overexpression exerted the opposite effect. In addition, we confirmed that MAL silencing substantially increased the metastasis and invasion of MGC-803 and HGC-27 cells (Fig. 3C–R), whereas MAL overexpression inhibited the metastasis and invasion of GC cells (Fig. 3C–R).

### MAL suppresses the epithelial-mesenchymal transition (EMT) process in GC cells

**Fig. 4:**
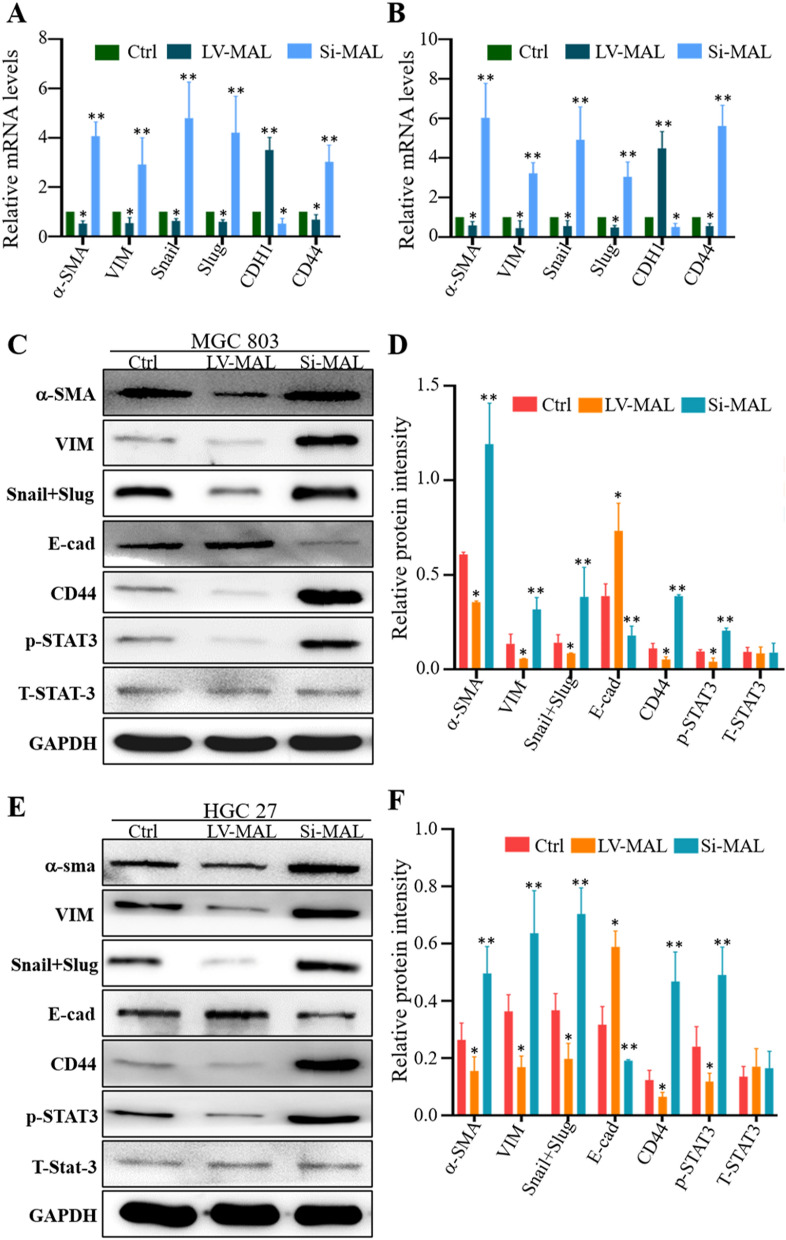
The MAL protein inhibits the EMT in GC cells. The RT–qPCR results showed that MAL silencing promoted the occurrence of the EMT in MGC-803 (**A**) and HGC-27 (**B**) GC cells, and MAL overexpression inhibited the EMT in MGC-803 (**A**) and HGC-27 (**B**) GC cells. MAL knockdown induced the occurrence of the EMT in MGC-803 (**C**) and HGC-27 cells (**E**), and MAL overexpression inhibited the EMT in MGC-803 (**C**) and HGC-27 (**E**) cells, as indicated by Western blotting. Statistical quantification of the expression of EMT-related proteins in the control group, MAL interference group and MAL overexpression group of MGC-803 (**D**) and HGC-27 (**F**) cells. The experiments were performed at least 3 times independently, and one representative result is shown. The results are presented as the means ± SD. *P < 0.05 and **P < 0.01

The EMT has been confirmed to occur in many cancer types because it leads to the loss of the epithelial phenotype and the acquisition of mesenchymal characteristics in tumor cells, which will lead to local invasion into surrounding tissues and systemic spread to distant organs [15]. Our results showed that MAL overexpression inhibited the expression of mesenchymal markers and promoted the expression of epithelial markers in GC cells (Fig. 4). However MAL knockdown promoted EMT-like changes (Fig. 4).

### MAL affects the EMT in GC cells by inhibiting STAT3 signaling

**Fig. 5:**
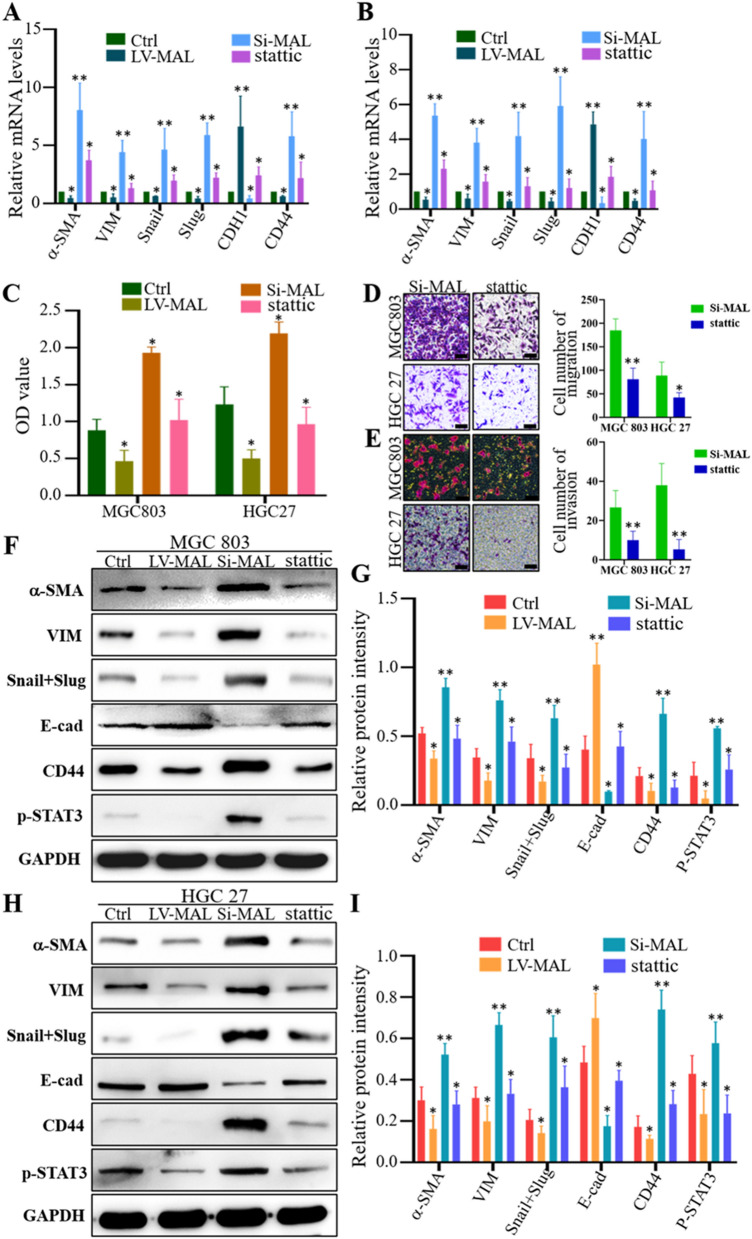
The MAL protein interferes with the EMT in GC cells by inhibiting the STAT3 pathway. The CCK-8 results showed that the inhibitor of STAT3 (Stattic, 10 μm) significantly attenuated the proliferation of MGC-803 and HGC-27 cells induced by MAL knockdown (**A**). Stattic treatment (10 μm) significantly attenuated the increases in the metastasis (**B**) and invasion (**C**) of MGC-803 and HGC-27 cells caused by MAL knockdown. RT–qPCR data showed that Stattic attenuated the MAL knockdown-induced increases in the mRNA expression of EMT markers in MGC-803 (**D**) and HGC-27 € cells. Western blotting showed that Stattic significantly attenuated the increases in EMT-related protein levels in MGC-803 (**F**) and HGC-27 (**H**) cells induced by MAL knockdown. Statistical quantification of the four groups of MGC-803 (**G**) and HGC-27 (**I**) cells was performed. The experiments were performed at least 3 times independently, and one representative result is shown. The results are presented as the means ± SD. *P < 0.05 and **P < 0.01. B and C, Scale bars = 100 μm

Studies have also confirmed that STAT3 is upregulated in most cancers and participates in promoting tumor invasion [16]. As shown in our previous studies, isoproterenol binds to β2 receptors to activate the STAT3 pathway and induce the EMT in GC cells [11, 14]. Because STAT3 regulates tumor progression, we next verified whether STAT3 was involved in MAL-mediated inhibition of the EMT in GC cells. The results in Fig. 3 confirmed that overexpression of MAL in MGC-803 and HGC-27 cells reduced p-STAT3 levels and that MAL silencing increased p-STAT3 levels (Fig. 4). Subsequently, the CCK-8 results showed that an inhibitor of STAT3 (Stattic) attenuated the increased proliferation of GC cells with MAL knockdown (Fig. 5A). In addition, Stattic also inhibited the increases in cancer cell metastasis and invasion after interfering with MAL expression (Fig. 5B, C). Furthermore, Stattic altered MAL knockdown-induced EMT activation in GC cells (Fig. 5E–I).

### MAL inhibits proliferation and promotes apoptosis of GC cells in a mouse tumorigenesis model

**Fig. 6:**
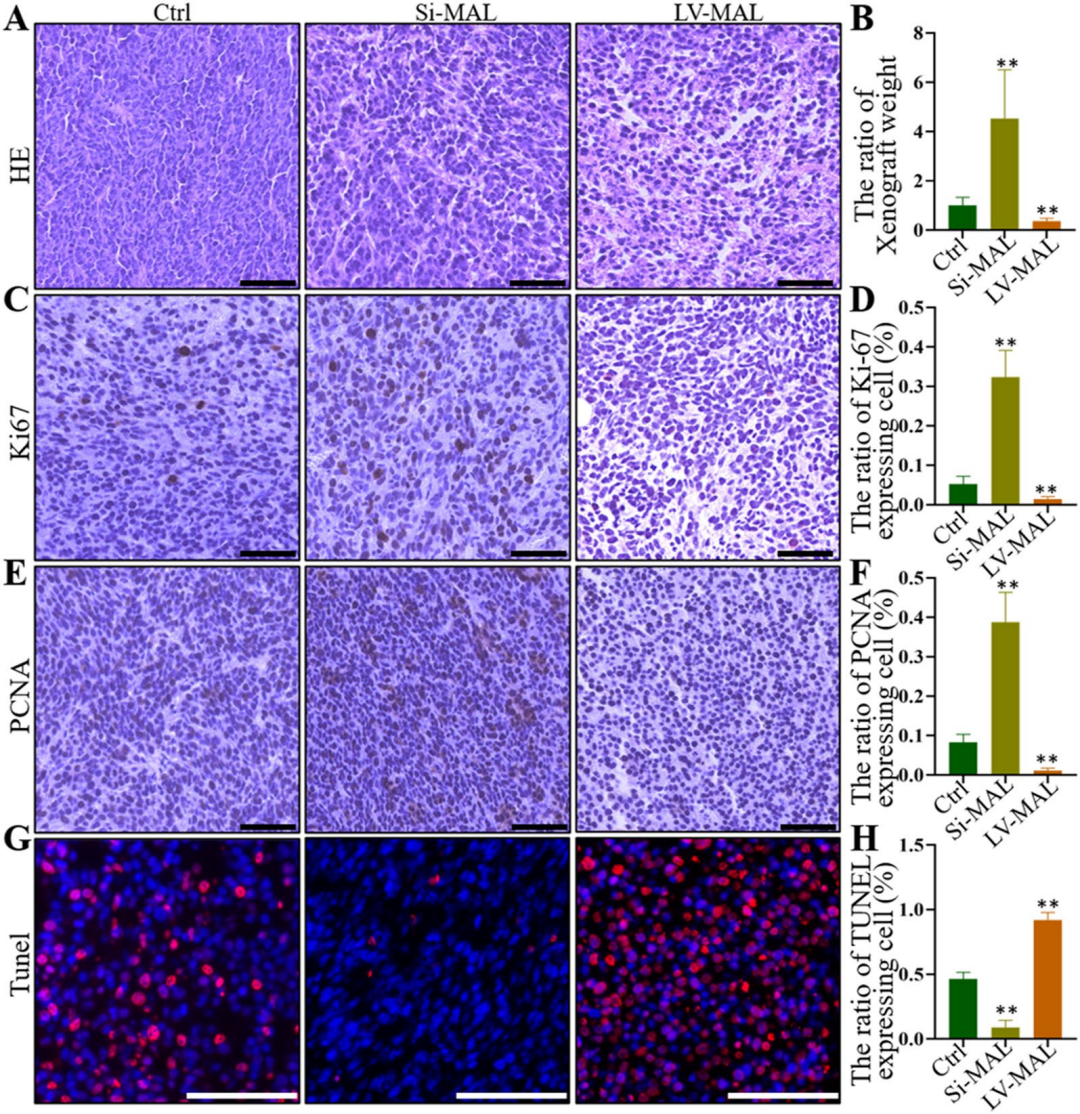
MAL inhibited the proliferation and promoted the apoptosis of GC cells in a mouse tumorigenesis model. MGC-803 cells with MAL knockdown or overexpression were injected subcutaneously at a dose of 10^7^ cells per site, and xenograft tumors were dissected and removed for histopathological analysis 14 days after injection. The bar graph shows the quantitative analysis of the weight of gastric tumors (**B**). HE staining was performed to analyze the pathological changes in the control, MAL knockdown, and MAL-overexpressing MGC-803 cells (**A**). Immunohistochemistry revealed the expression of Ki-67 in control, MAL knockdown, and MAL-overexpressing MGC-803 cells (**C**). Immunohistochemistry revealed the expression of PCNA in control, MAL knockdown, and MAL-overexpressing MGC-803 cells (**E**). The TUNEL assay was used to verify cell apoptosis in control, MAL knockdown, and MAL-overexpressing xenograft tumor tissues (**G**). The bar graph shows the proportion of cells with positive staining for Ki-67 (**D**), PCNA (**F**) and TUNEL (**H**). The experiments were performed at least 3 times independently, and one representative result is shown. The results are presented as the means ± SD. *P < 0.05 and **P < 0.01. A–C, F–G, I–K and M–O, Scale bars = 100 μm

We injected GC cells with MAL knockdown or overexpression into the backs of nude mice to further confirm the role of MAL in GC progression. After 15 days of rearing, the tumor tissues were removed from the backs of nude mice, and we assessed cell proliferation and apoptosis. Immunohistochemical data showed that in the group engrafted with MAL knockdown cells, the number of cells with positive expression of cell proliferation markers (Ki-67 and PCNA) increased significantly, but the opposite effect was observed on the overexpression group (Fig. 6A–H). In addition, the results of TUNEL staining revealed significantly fewer positive cells in the MAL knockdown group, while the overexpression group showed more positive cells than the control group (Fig. 6M–P).

## Discussion

GC is a malignant tumor with an increasing incidence and a poor patient prognosis [17], indicating that it is still an unresolved clinical problem. The acute effect of GC on the population of most developed and developing nations is much more serious [18]. We explored the molecular mechanism of GC progression and identified the MAL/STAT3/EMT regulatory axis in GC cells. MAL mediates the mesenchymal transition of GC cells by inhibiting the STAT3 pathway, thereby regulating the proliferation, metastasis and invasion of tumor cells. MAL may be a new treatment target and molecular diagnostic marker for GC.

**Fig. 7:**
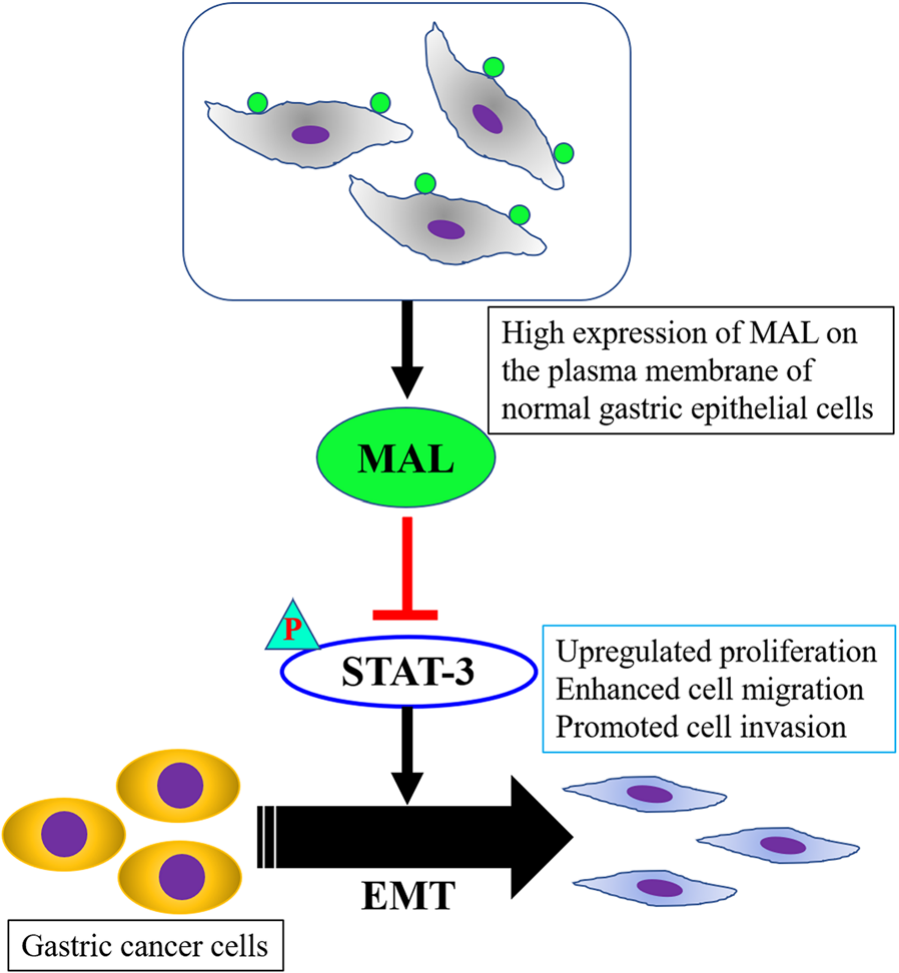
The MAL protein is expressed at high levels in normal gastric tissues but expressed at low levels in primary GC tumors and metastases. We further proved that MAL participates in inhibiting the proliferation, metastasis and invasion of GC cells. MAL inhibits STAT3, mediates the EMT, and subsequently inhibits human GC progression

MAL is an indispensable membrane protein in lipid rafts that participates in the apical transport of proteins in polarized epithelial cells, and the role of its abnormal expression in the occurrence and development of tumors has received increasing attention [19]. In this study, we assessed the MAL expression pattern in pericancerous and primary gastric tumor samples. MAL was expressed at high levels in normal gastric samples and very low levels in the primary tumor and corresponding metastases. MAL did not have the same expression levels in different types of normal gastric tissue cells. Interestingly, MAL was mainly expressed in epithelial cells of gastric mucosa. In addition, the interaction between MAL and glycosphingolipids plays an important role in the formation of microdomains in gastric epithelial cells. Because CD44 represents the stemness of GC cells, we performed costaining for CD44 and MAL in pericancerous and primary GC samples. Our results indicated opposite expression patterns for MAL and CD44 in matched primary tumor samples, indicating a negative correlation in vivo. These studies fully illustrate an essential role for MAL in maintaining the normal function of the stomach. We verified the function of MAL in the GC process using two human GC cell lines and found that MAL hinders the proliferation, metastasis and invasion of cancer cells. Notably, we observed that MAL silencing enhanced the EMT of GC cells. Studies have found that the EMT is closely related to the occurrence and development of GC [20]. GC cells activate the EMT through complex molecular mechanisms and numerous signaling pathways to modulate their own proliferation, invasion and migration [21]. STAT3 plays a key role in signal transduction pathways of cancer stem cells [22], and we found that MAL overexpression inhibited STAT3 phosphorylation in GC cells and MAL knockdown increased STAT3 phosphorylation. Using pharmacological inhibitors to analyze the proliferation, metastasis and invasion of cancer cells and the expression of EMT-related markers, we further showed that MAL inhibited the proliferation, metastasis and invasion of GC cells and interfered with the EMT by inhibiting STAT3 (Fig. 7).

The more controversial finding is the involvement of MAL in carcinogenesis through two opposing pathways. First, MAL is overexpressed in ovarian cancer [23] and certain types of lymphomas [24–26] and appears to promote tumor progression. In contrast, MAL promoter hypermethylation and concomitant MAL downregulation have been described in other epithelial malignancies, such as colon [27], breast [28], salivary gland cancers [29], non-small cell lung cancer (NSCLC) [8] and bladder cancer [30]. Buffart and Choi documented frequent hypermethylation of the MAL promoter in GC that was associated with downregulation of its expression [31, 32]. Methylation of this region within the MAL promoter is associated with significantly prolonged patient survival and may serve as an independent prognostic marker of clinical outcomes in patients with GC [31]. MAL is involved in the apical transport of proteins in polarized epithelial cells; however, apart from the role of MAL in apical sorting and its function as a raft stabilizer, researchers have not clearly determined the mechanism by which MAL regulates the development of tumor cells. Here, we identified a surprising role for MAL in regulating the proliferation, metastasis and invasion of GC cells. The proliferation, metastasis and invasion of cancer cells are the main factors contributing to the poor treatment effect on patients with advanced GC. Preventing the metastasis, invasion and abnormal proliferation of GC cells and improving the survival rate of these patients are currently important topics in GC research. The proliferation, invasion and metastasis of GC cells is a complex multistage process that is regulated by multiple genes, factors and signaling pathways. STAT3 is proposed to function as an oncogene in a variety of malignant tumors and participate in signal transduction in tumor stem cells. Our results showed that MAL overexpression reduced p-STAT3 levels in GC cells, but the expression of T-STAT3 was not substantially affected. Therefore, we speculate that it is involved in the progression of GC, inhibits the expression of the MAL protein, and then activates its downstream target STAT3. STAT3 activation promotes the EMT, thereby increasing the proliferation, metastasis and invasion of GC cells. Therefore, in-depth explorations of the functional regulatory mechanism of GC cells, clarification of the pathogenesis of GC, and identification of new therapeutic targets may provide new target genes for the treatment of GC and other human malignancies.

## Conclusions

In conclusion, we further determined that MAL inhibits the malignant behavior of GC cells through the STAT3/EMT axis. MAL provides insights into the pathophysiological process of GC, and its tumor suppressor function may be expected to become a novel therapeutic target in GC.

## Data Availability

Not applicable.
